# Supplementary material for: Electron–Phonon Coupling and Electron–Phonon Scattering in SrVO3
Source: Adv Sci (Weinh). 2021 Jun 19;8(15):2004207. doi: 10.1002/advs.202004207 (PMC8336622; doi:10.1002/advs.202004207)
Supplement: Supplementary file 1 — Supporting Information [file ADVS-8-2004207-s001.pdf]

WILEY-VCH

© 2021 Wiley-VCH GmbH

ADVANCED SCIENCE
Open Access

Supporting Information

for *Adv. Sci.*, DOI: 10.1002/advs.202004207

Electron-Phonon Coupling and Electron-Phonon Scattering in $SrVO_3$

*Mathieu Mirjolet, Francisco Rivadulla, Premysl Marsik, Vladislav Borisov, Roser Valentí, and Josep Fontcuberta**

Supporting Information

Electron-Phonon Coupling and Electron-Phonon Scattering in $SrVO_3$

*Mathieu Mirjolet, Francisco Rivadulla, Premysl Marsik, Vladislav Borisov, Roser Valentí, and Josep Fontcuberta**

Supporting Information S1: Structural and topographic morphological data.

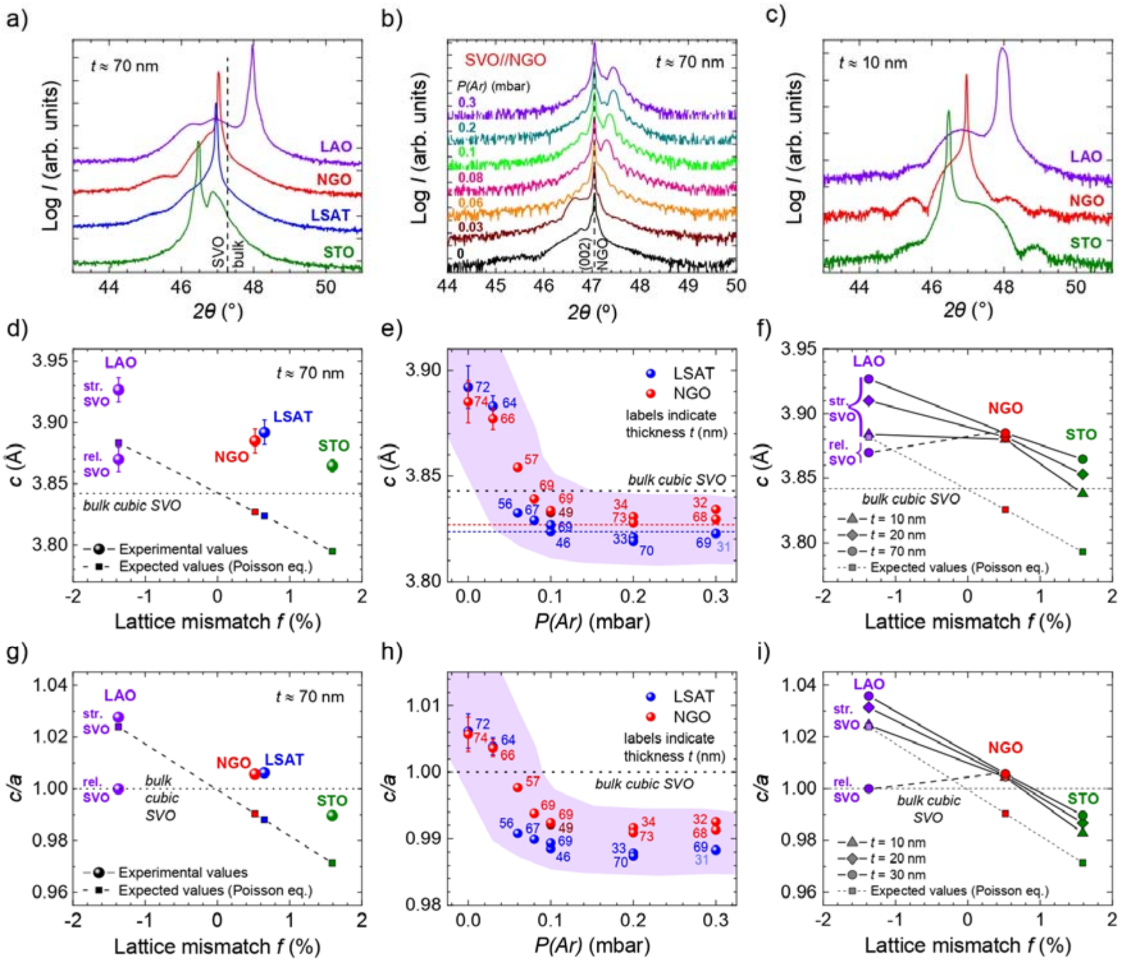

Figure S1a: (a-c) Illustrative X-ray diffraction patterns for most of SVO films of this study. From left to right column: strain series (different substrates, at constant thickness of about 70 nm), *P(Ar)* series on LSAT and NGO, and thickness series (10, 20 and 70 nm) on various substrates. (d-f) Extracted *c*-axis parameters for the corresponding samples. To minimize the measurement error, the *c*-axis was extrapolated by Nelson-Riley method using the (00*l*) family ($l = \{1; 2; 3; 4\}$) of diffraction peaks. From our reciprocal space maps[1] all films deposited on

NGO, LSAT and STO (tensile strain) were fully strained ($a_{SVO} = a_S$). Thick SVO films (70 nm) deposited on LAO (compressive strain) show strain relaxation, while thinner ones (10-20 nm) are nearly fully strained. (g-i) Corresponding tetragonal distortion (*c/a* ratio).

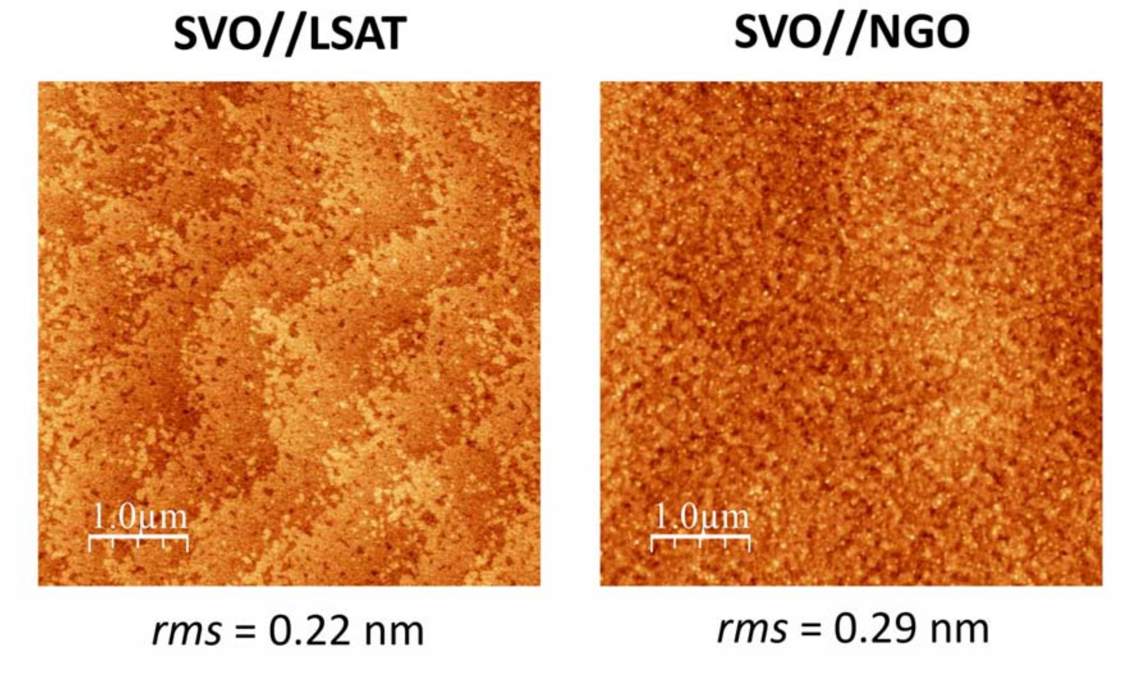

Figure S1b: Illustrative AFM topographic images of SVO films grown on LSAT (left) and NGO (right), at *P(Ar)* = 0.03 mbar. Films were about 70 nm thick. Image size is 5 μm x 5 μm.

Supporting Information S2: Carrier density, carrier mobility, and residual resistivity ratio.

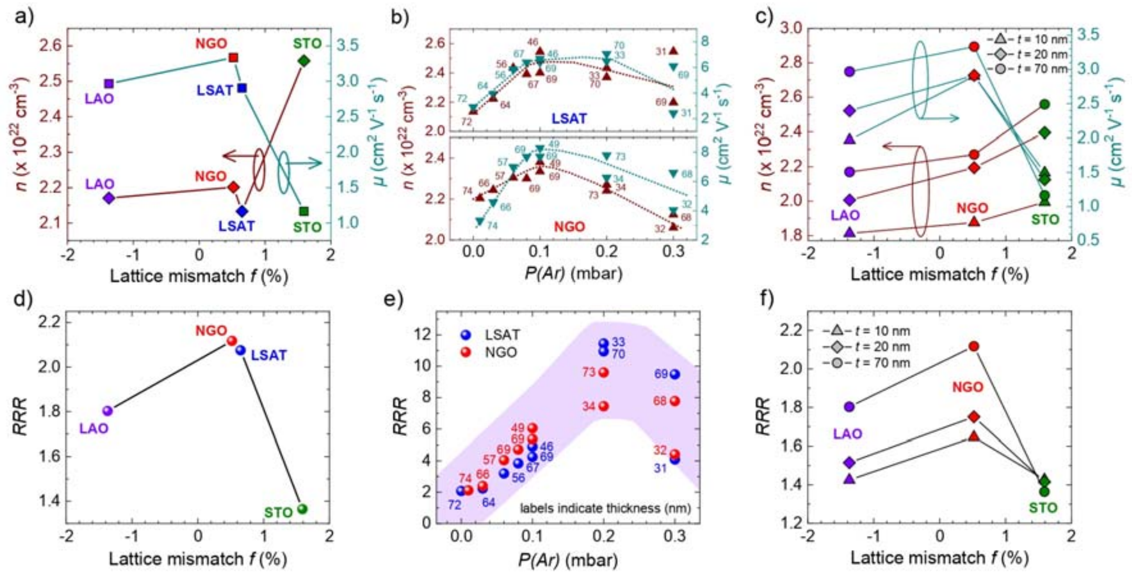

Figure S2: Transport data for most SVO films of this study. From left to right column: strain series (different substrates), *P(Ar)* series on LSAT and NGO, and thickness series (10, 20 and 70 nm) on various substrates. Upper panels show the room-temperature carrier density n and mobility μ. Lower panels show residual resistivity ratio (*RRR*) of the corresponding samples.

Supporting Information S3: Illustrative Hall effect measurements.

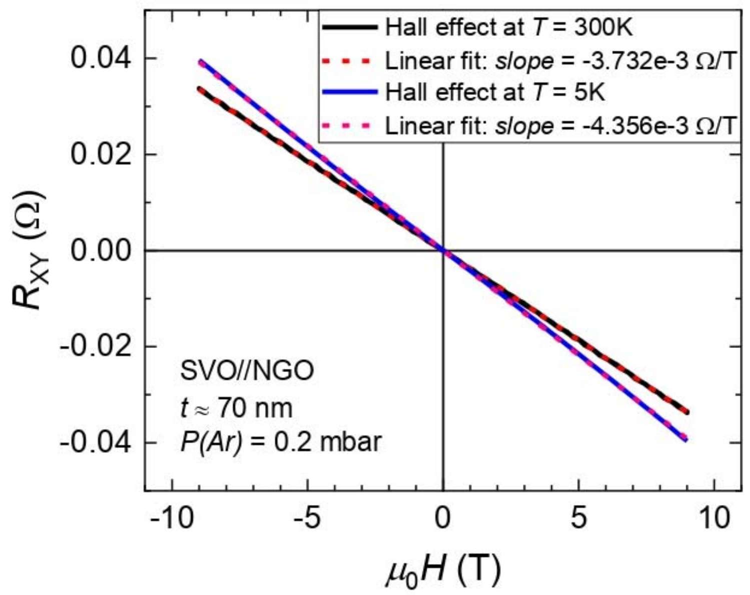

Figure S3: Illustrative Hall effect measurements at 300 K and 5 K of a 70 nm thick SVO film deposited on NGO substrate, at *P(Ar)* = 0.2 mbar.

Supporting Information S4: Fits of resistivity data to a quadratic temperature dependence and polaronic models.

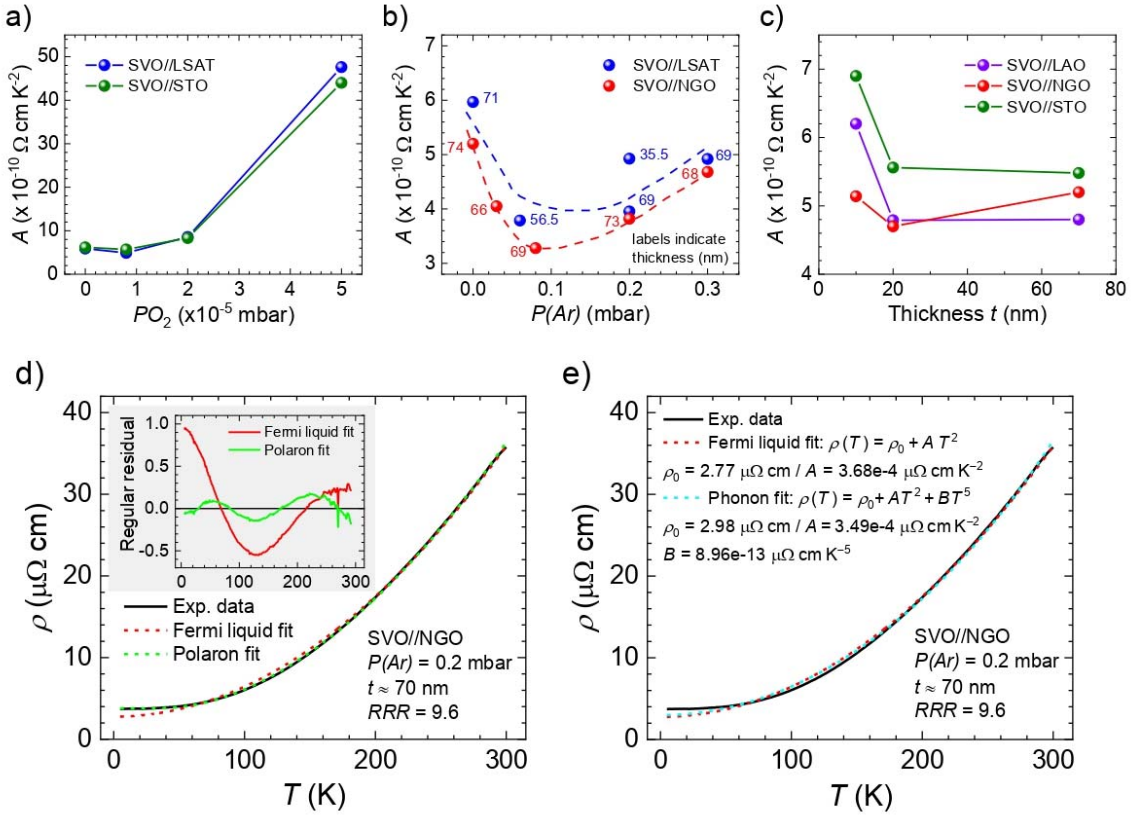

Figure S4a: Compilation of A values extracted from the fitting function $\rho(T) = \rho_0 + A\,T^2$. a) PO_2 series on STO and LSAT substrates. b) *P(Ar)* series on LSAT and NGO substrates. c) Thickness series on LAO, NGO and STO substrates. d) Illustrative results of the fit of $\rho(T)$ using the T^2-dependence and the polaronic model. Inset show the residual difference $[\rho_{\mathrm{exp}} - \rho_{\mathrm{fit}}]$. Notice that the residual difference at $T < 180\text{-}200$ K becomes about a factor 10 larger in the quadratic model. e) Comparison of the T^2 and $A\,T^2 + B\,T^5$ fits together with the polaronic model, for an illustrative film.

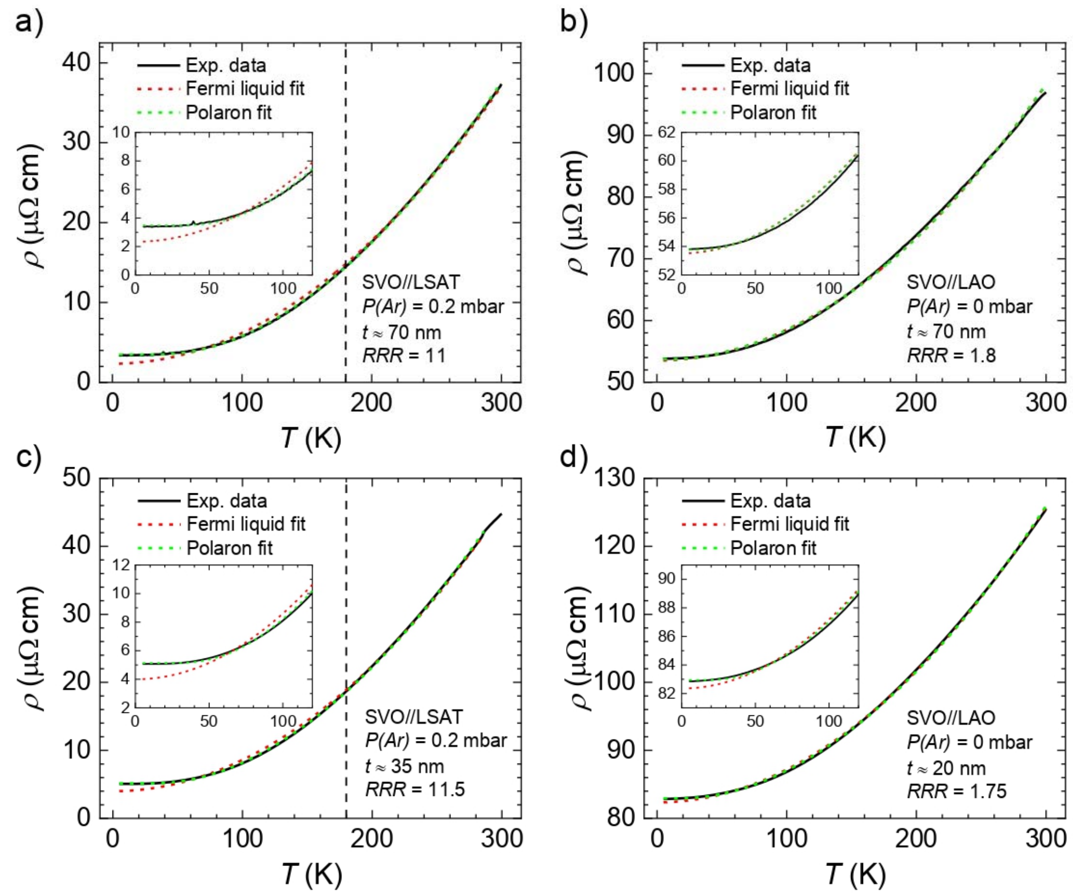

Figure S4b: Additional $\rho(T)$ data of SVO films together with Fermi liquid fit (red dashed curve) and polaron model fit (green dashed curve). Left panels show SVO of thickness: a) 70 nm and c) 35 nm thick film; deposited on LSAT at *P(Ar)* = 0.2 mbar. Right panels show SVO film of thickness: b) 70 nm and d) 20 nm; deposited on LAO at *P(Ar)* = 0 mbar. Insets are zooms of the low temperature region where the Fermi liquid fits show highest discrepancy.

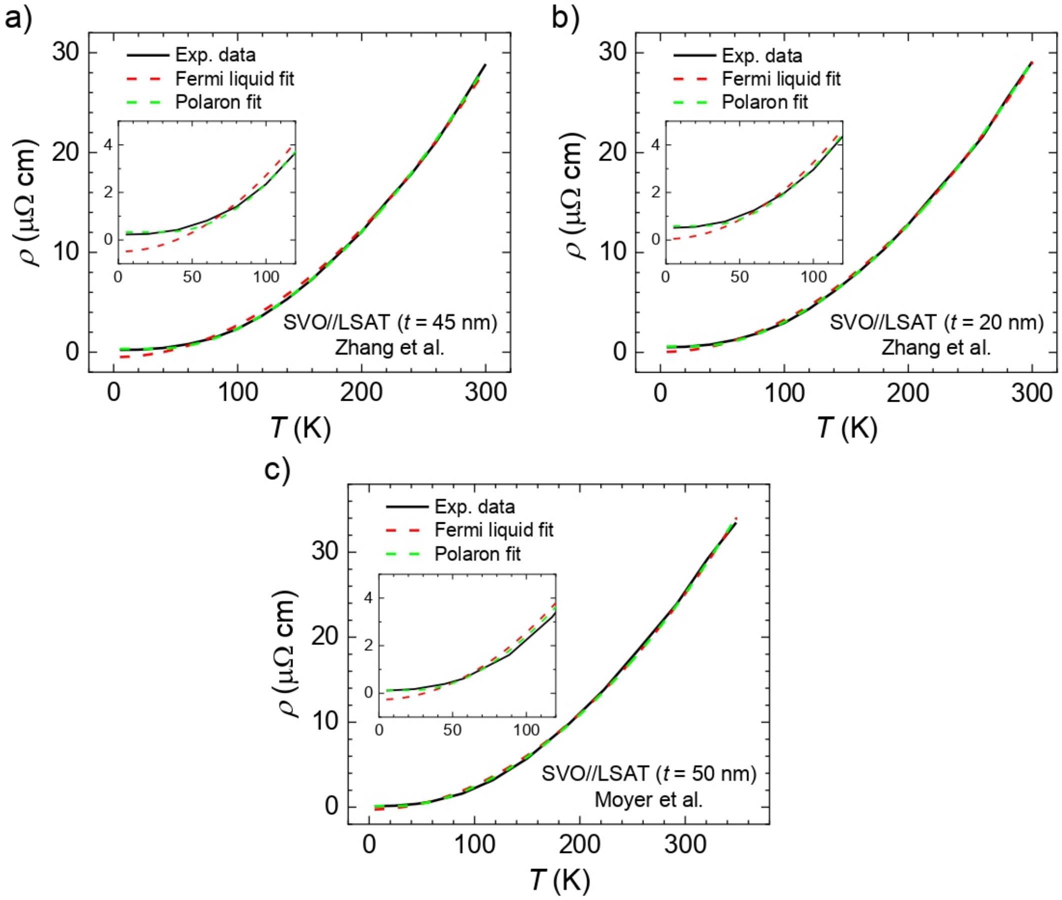

Figure S4c: Literature $\rho(T)$ data of SVO films grown on LSAT by hybrid-MBE, together with Fermi liquid fit (red dashed curve) and polaron model fit (green dashed curve). Data in (a,b) represent digitized data of 45 and 20 nm thick films, respectively, from Zhang et al.[2] Data in (c) are taken from Moyer et al.[3] (50 nm thick). Insets are zooms of the low temperature region where the Fermi liquid fits show highest discrepancy.

Fitting parameters:

Table S4-I: Fitting parameters for Fermi liquid (constrained and unconstrained) and polaronic fits, for some illustrative SVO films: SVO films (10 nm) on STO, NGO and LAO (data are shown in Figure 5). Notice the errors of parameters are < 6 %.

Sample	Fit	R^2	A_{ee} [Ω cm K^{-2}]	A^*_{e-ph} [Ω m s]	$\hbar\omega_0$ [meV]
SVO//STO (Fig. 5c)	Fermi liquid (fixed ρ_0)	0.99**314**	6.35e-10 ± 2.14e-12	x	x
	Fermi liquid	0.99**860**	6.72e-10 ± 1.46e-12	x	x
	Polaronic	0.99**995**	x	1.20e-20 ± 1.29e-23	20.6 ± 0.11
SVO//NGO (Fig. 5d)	Fermi liquid (fixed ρ_0)	0.99**867**	4.955e-10 ± 7.12e-13	x	x
	Fermi liquid	0.99**962**	5.07e-10 ± 5.74e-13	x	x
	Polaronic	0.99**980**	x	9.78e-21 ± 2.77e-22	12.74 ± 0.35
SVO//LAO (Fig. 5e)	Fermi liquid (fixed ρ_0)	0.99**899**	9.04e-10 ± 1.12e-12	x	x
	Fermi liquid	0.99**954**	9.20e-10 ± 1.15e-12	x	x
	Polaronic	0.99**962**	x	8.30e-21 ± 4.58e-22	10.75 ± 0.58

Table S4-II: Comparison of fitted parameters depending on input parameters for the polaronic fit. It can be appreciated that the input parameters can varied by about 3 orders of magnitude but the fitted values are virtually identical.

Sample	Initial $\hbar\omega_0$ [meV]	Initial A^*_{e-ph} [Ω m s]	Reduced chi-squared	R^2	$\hbar\omega_0$ [meV]	Dependency	A^*_{e-ph} [Ω m s]	Dependency
SVO//NGO (Fig. 5d)	**10**	**1e-20**	**0.0374**	**0.9998**	**12.74**	**0.9996**	**9.78e-21**	**0.9996**
	100	1e-20	0.0374	0.9998	12.74	0.9996	9.78e-21	0.9996
	1	1e-20	0.0374	0.9998	12.74	0.9996	9.78e-21	0.9996
	10	1e-19	0.0374	0.9998	12.74	0.9996	9.78e-21	0.9996
	10	1e-21	0.0374	0.9998	12.74	0.9996	9.78e-21	0.9996
	100	1e-19	0.0374	0.9998	12.74	0.9996	9.78e-21	0.9996
	100	1e-21	0.0374	0.9998	12.74	0.9996	9.78e-21	0.9996
	1	1e-19	0.0374	0.9998	12.74	0.9996	9.78e-21	0.9996
	1	1e-21	0.0374	0.9998	12.74	0.9996	9.78e-21	0.9996

Supporting Information S5: Experimental procedure and complementary Seebeck coefficient data.

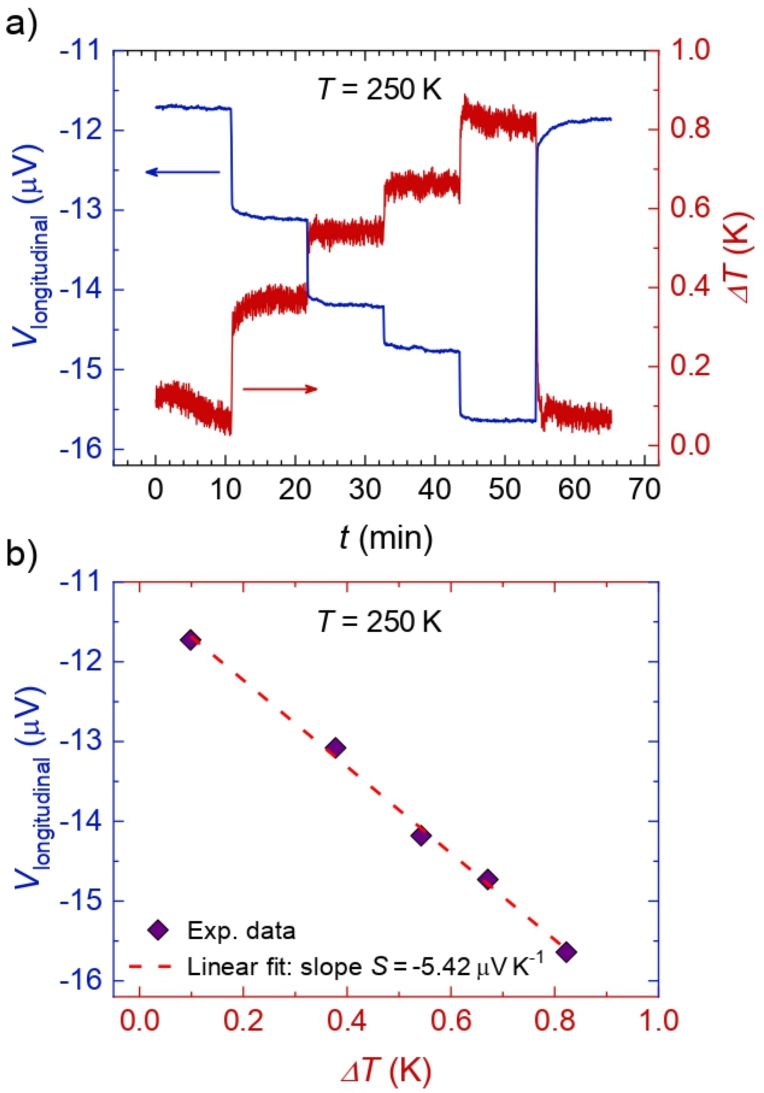

Figure S5a: Experimental details of the Seebeck measurements: a) Steps of temperature difference between the Pt resistances and corresponding longitudinal thermoelectric voltages, at a base temperature of 250 K, for one of the SVO films of this study. b) Linear fit of the longitudinal voltage vs temperature difference. The accuracy of the method allows a good measurement of the Seebeck coefficient without increasing much the temperature difference (always lower than 1.5 K), ensuring the reversibility of the process. In this example a Seebeck coefficient of $S = -5.42\ \mu V\ K^{-1}$ at $T = 250$ K was extracted. The whole temperature dependence of the Seebeck coefficient for this sample can be seen in Figure S5b (SVO//LSAT, blue curve).

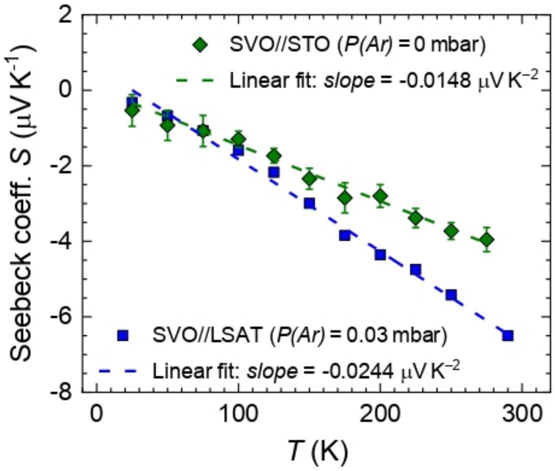

Figure S5b: Temperature dependence of the Seebeck coefficient S measured on additional SVO films ($t \approx 70$ nm thick), deposited on LSAT and STO substrates, having different carrier density $n = 2.13\text{x}10^{22}$ cm^{-3} and $n = 2.56\text{x}10^{22}$ cm^{-3}, respectively.

Supporting Information S6: Softening of selected optical phonon modes with increasing tetragonality in SVO.

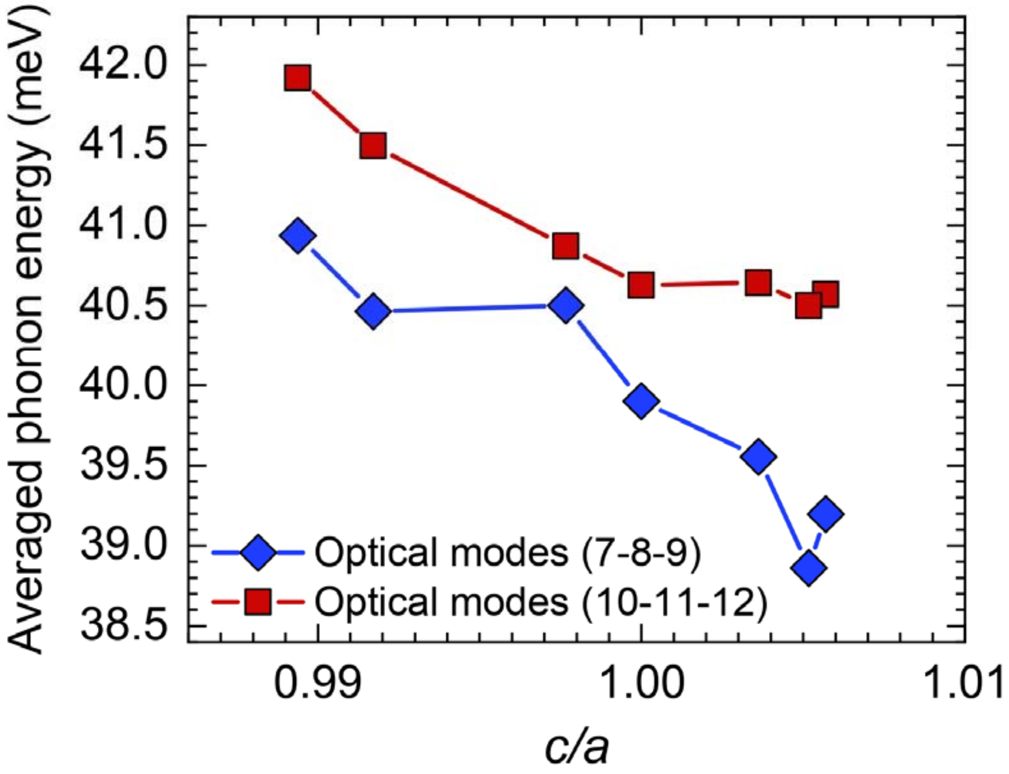

Figure S6: Phonons in SVO. Phonon energies at Gamma-point of SVO have been calculated as a function of *c/a* using density functional perturbation theory available in Quantum Espresso. Preliminary data for phonons displaying the strongest e-phonon coupling are shown. The (7-12) indexes refer to optical modes of increasing energy. As the modes 7, 8 and 9, as well as the modes 10, 11 and 12, are split due to the tetragonal distortion, here we plot the averaged values for each group of modes.

Supporting Information S7: Ellipsometric data.

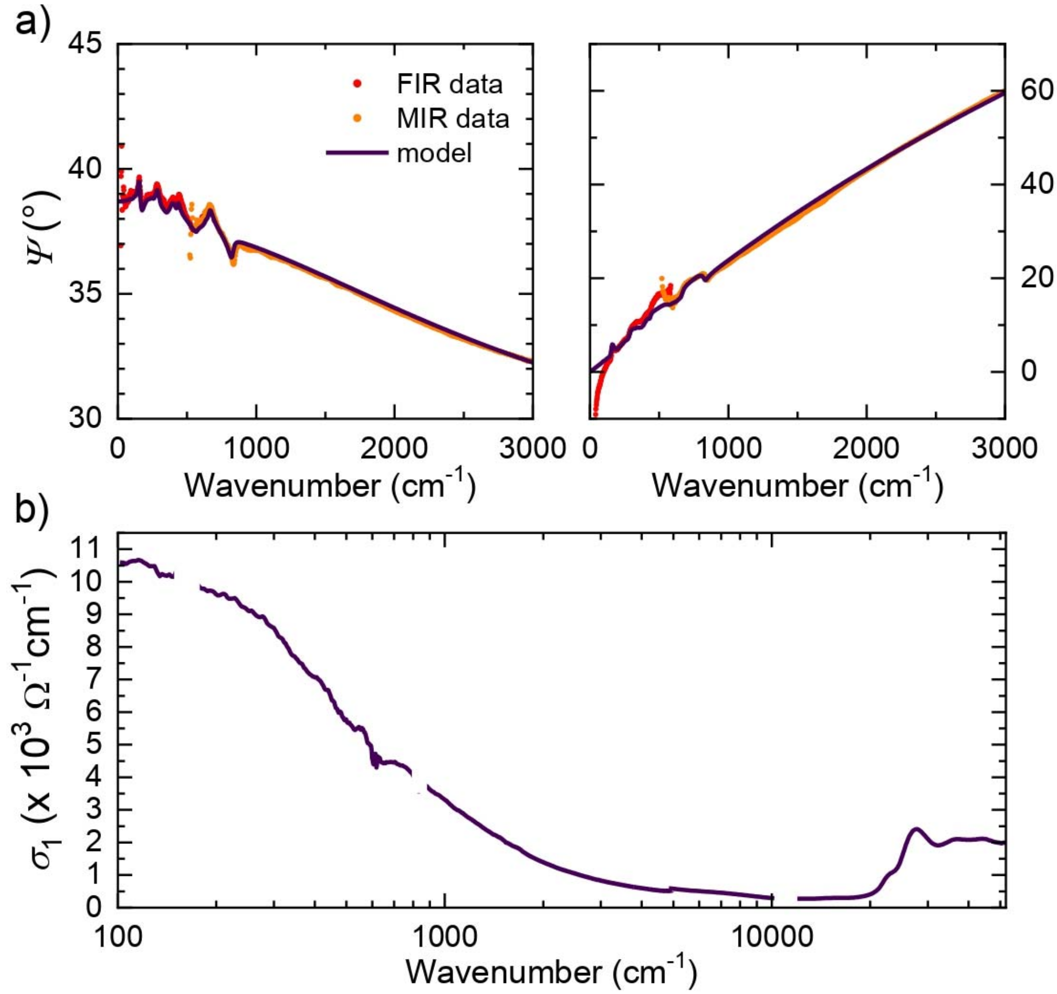

Figure S7: a) Ellipsometric data (FIR + MIR) of a SVO film (72 nm thick) deposited on LSAT substrate. The measured (Ψ, Δ) spectra are fitted with a substrate/film/ambient model, where only the film response is varied. Substrate response was determined from ellipsometric measurements on a bare substrate.[4] The far-infrared response of the film is dominated by a Drude component. The extracted unscreened plasma frequency is $\omega_p = 19500\ \mathrm{cm}^{-1}$ (≈ 2.42 eV) and broadening $\gamma = 680\ \mathrm{cm}^{-1}$, which corresponds to a screened plasma energy $E^*_{\omega_p} = 1.21$ eV (considering $\varepsilon_\infty = 4$, as reported by Makino et al.[5]). The corresponding effective mass $m^* \approx 4.1$ m_e. Notice that we reported similar values in our previous studies,[1,6] and that similar values were encountered in literature.[2] Features in the Ψ/Δ spectra below $1000\ \mathrm{cm}^{-1}$ originate from the substrate phonons. b) Optical conductivity σ_1 of the SVO film up to UV (6.2 eV, 50000 cm^{-1}) resulting from point-by-point fit to ellipsometry data.

Supporting Information S8: Extended universal scaling between the prefactor of the T^2 dependent resistivity and the Fermi energy.

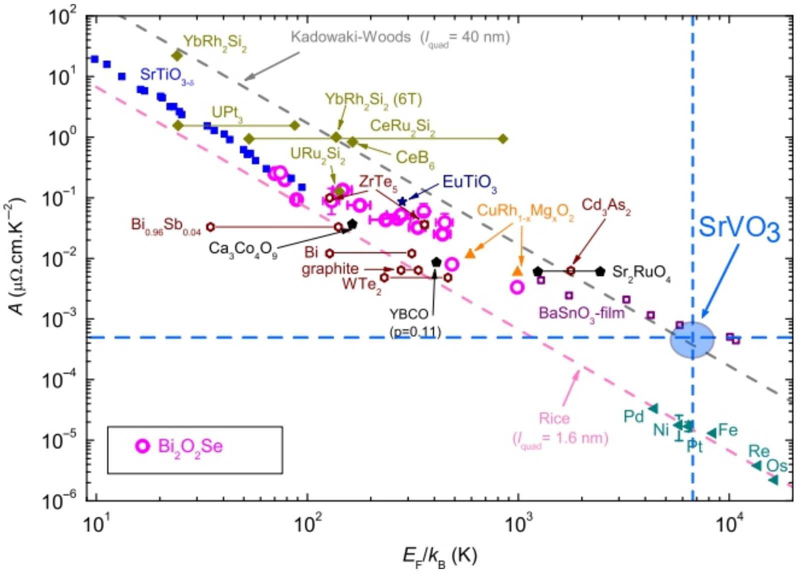

Figure S8: Scaling between the prefactor of the T^2 dependent resistivity and the Fermi energy. Our data for SVO are plotted on top of data taken from Wang et al.[7]

References

[1] M. Mirjolet, H. B. Vasili, Ll. López-Conesa, S. Estradé, F. Peiró, J. Santiso, F. Sánchez, P. Machado, P. Gargiani, M. Valvidares, J. Fontcuberta, *Adv. Funct. Mater.* **2019**, *29*, 1904238.

[2] L. Zhang, Y. Zhou, L. Guo, W. Zhao, A. Barnes, H.-T. Zhang, C. Eaton, Y. Zheng, M. Brahlek, H. F. Haneef, N. J. Podraza, M. H. W. Chan, V. Gopalan, K. M. Rabe, R. Engel-Herbert, *Nat. Mater.* **2016**, *15*, 204.

[3] J. A. Moyer, C. Eaton, R. Engel-Herbert, *Adv. Mater.* **2013**, *25*, 3578.

[4] T. N. Nunley, T. I. Willett-Gies, J. A. Cooke, F. S. Manciu, P. Marsik, C. Bernhard, S. Zollner, *J. Vac. Sci. Technol. A* **2016**, *34*, 051507.

[5] H. Makino, I. Inoue, M. J. Rozenberg, I. Hase, Y. Aiura, S. Onari, *Phys. Rev. B* **1998**, *58*, 4384.

[6] M. Mirjolet, F. Sánchez, J. Fontcuberta, *Adv. Funct. Mater.* **2019**, *29*, 1808432.

[7] J. Wang, J. Wu, T. Wang, Z. Xu, J. Wu, W. Hu, Z. Ren, S. Liu, K. Behnia, X. Lin, *Nat. Commun.* **2020**, *11*, 3846.
